# Opportunistic Screening Techniques for Analysis of CT Scans

**DOI:** 10.1007/s11914-022-00764-5

**Published:** 2022-11-26

**Authors:** Klaus Engelke, Oliver Chaudry, Stefan Bartenschlager

**Affiliations:** 1 Department of Medicine III, Friedrich-Alexander University of Erlangen-Nürnberg, University Hospital Erlangen, Ulmenweg 18, 91054 Erlangen, Germany; 2 Institute of Medical Physics (IMP), Friedrich-Alexander-Universität Erlangen-Nürnberg (FAU), Henkestr. 91, 91052 Erlangen, Germany

**Keywords:** Computed tomography, Opportunistic screening, Fracture risk, Vertebral fracture assessment, Internal BMD calibration

## Abstract

**Purpose of Review:**

Opportunistic screening is a combination of techniques to identify subjects of high risk for osteoporotic fracture using routine clinical CT scans prescribed for diagnoses unrelated to osteoporosis. The two main components are automated detection of vertebral fractures and measurement of bone mineral density (BMD) in CT scans, in which a phantom for calibration of CT to BMD values is not used. This review describes the particular challenges of opportunistic screening and provides an overview and comparison of current techniques used for opportunistic screening. The review further outlines the performance of opportunistic screening.

**Recent Findings:**

A wide range of technologies for the automatic detection of vertebral fractures have been developed and successfully validated. Most of them are based on artificial intelligence algorithms. The automated differentiation of osteoporotic from traumatic fractures and vertebral deformities unrelated to osteoporosis, the grading of vertebral fracture severity, and the detection of mild vertebral fractures is still problematic. The accuracy of automated fracture detection compared to classical radiological semi-quantitative Genant scoring is about 80%. Accuracy errors of alternative BMD calibration methods compared to simultaneous phantom-based calibration used in standard quantitative CT (QCT) range from below 5% to about 10%. The impact of contrast agents, frequently administered in clinical CT on the determination of BMD and on fracture risk determination is still controversial.

**Summary:**

Opportunistic screening, the identification of vertebral fracture and the measurement of BMD using clinical routine CT scans, is feasible but corresponding techniques still need to be integrated into the clinical workflow and further validated with respect to the prediction of fracture risk.

## Introduction

Opportunistic screening (OS) denotes a technique to extract information from an existing image or stack of images such as a computed tomography (CT) or magnetic resonance (MR) scan originally obtained for a clinical purpose unrelated to this information. In a narrower sense used in the context of this review, opportunistic screening denotes the use of existing CT scans to identify subjects at high risk for osteoporotic fracture. Opportunistic screening has been associated with the following eight important promises: (a) the elderly population can be screened for two important fracture risk factors: prevalent fractures that are associated with a high risk of subsequent, in particular, osteoporotic vertebral fractures and the measurement of bone mineral density (BMD), typically of the spine or the hip, which is a risk factor for all future osteoporotic fractures. (b) These two risk factors can be determined using almost all chest / abdomen / pelvis CT scans obtained in the clinic, (c) virtually for free, and (d) without added radiation exposure. (e) Techniques of artificial intelligence (AI) can be used for automatic recognition of spinal fractures and perhaps even for the determination of BMD. (f) CT values measured in Hounsfield units can be used instead of calibrated BMD values. (g) Standardization of the CT protocol is not required and (h) application of contrast agents has little impact.

In reality, existing or new CT scans obtained for a diagnosis other than osteoporosis show a number of shortcomings that require a careful review of the eight promises above: often only a part of the spine is imaged whereas the standard X-ray-based vertebral fracture assessment covers the range from T4 to L5. BMD is usually determined by quantitative CT (QCT) [1, 2], a procedure that uses an in-scan calibration phantom to convert CT to BMD values, and acquisition and reconstruction parameters are highly standardized. Application of contrast agents is not allowed in QCT. In OS most or all of these QCT conditions are violated. Thus, in order to use routine CT scans for risk assessments in osteoporosis, a number of new techniques were developed.

In this contribution we will review the published literature with respect to vertebral fracture assessment and determination of BMD from CT scans in the context of opportunistic screening. Chest, abdominal and pelvic CT scans are among the most frequent clinical CT scans that can be exploited. The value of OS in osteoporosis has been recognized already a couple of years ago as the identification of subjects with a high fracture risk promises a considerable reduction of the burden of osteoporotic fracture. Osteoporosis is mostly a disease of subjects above 50 years of age, for which also the frequency of CT scans increases rapidly.

## Vertebral Fracture Assessment

**Fig. 1:**
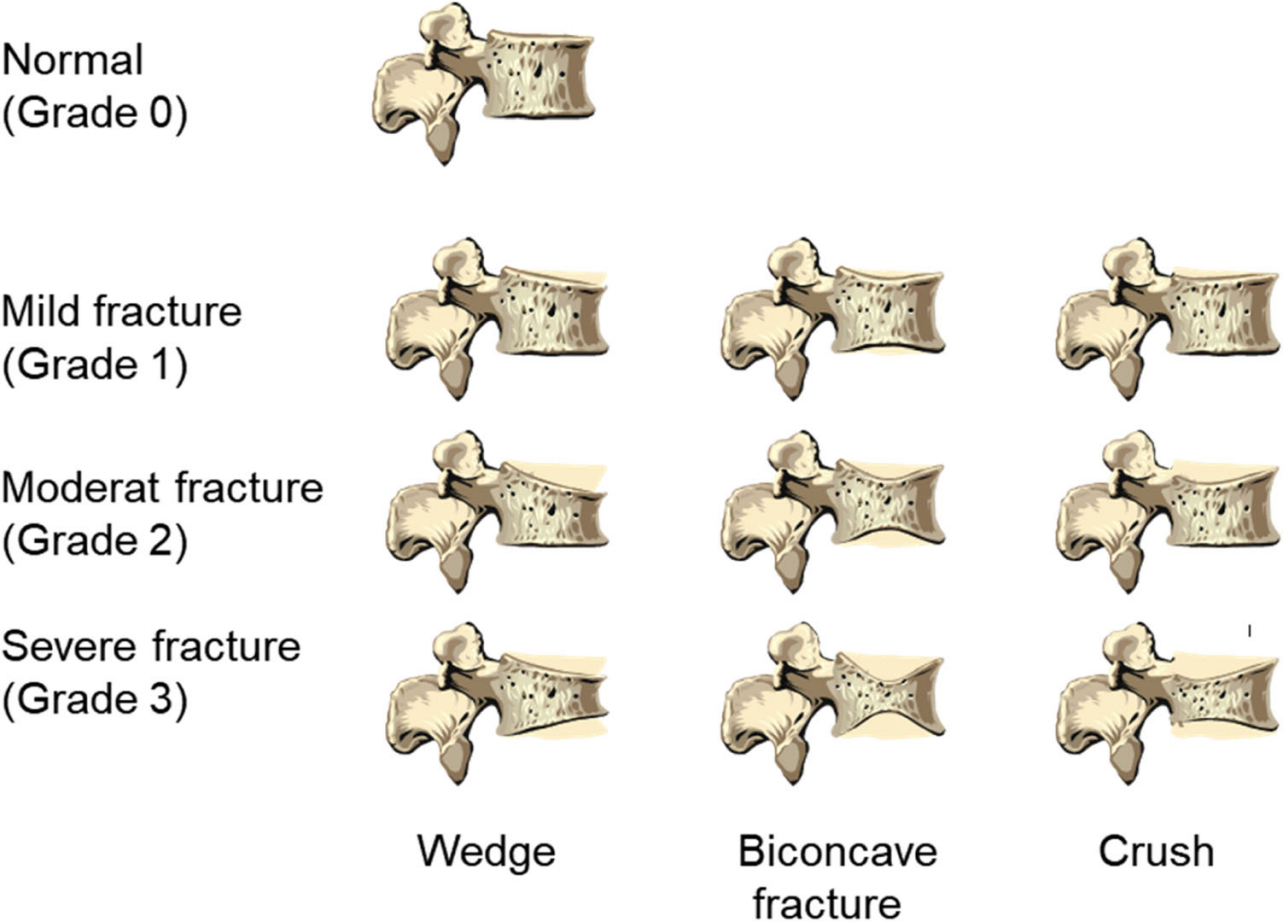
Genant scheme for semi-quantitative fracture assessment [3]

The most widely used technique for vertebral fracture assessment in osteoporosis is the semi-quantitative Genant scoring technique, which historically has been applied to lateral and posterior-anterior spine X-ray films [3] (Fig. 1). Outcome is the grade of osteoporotic fracture. The reading process implicitly includes the differentiation of osteoporotic from traumatic fractures and from vertebral deformities unrelated to osteoporosis, which do not increase fracture risk [4]. A broad range of techniques mostly based on automated image processing have been applied for vertebral fracture assessment ranging from identification of vertebrae with a high likelihood of fracture to 3D shape analysis and Genant equivalent scores including segmentation for subsequent automated BMD measurements [5] (Table 1). Obviously, these techniques have not been developed specifically for OS. Table 1 references a selected set of studies using CT. Several algorithms for automatic vertebral fracture assessment have also been developed for other imaging modalities such a DXA [37, 38] and X-rays [39, 40].

**Table 1:** Techniques for automatic vertebral fracture assessment and for vertebral segmentation

	Refs	Description
AI based techniques		
Heat Map	[6]	Heat maps are color-coded probability maps overlaid on the input image to visualize the outcome such as vertebral fracture identification and localization of a neural network. Such an image may assist the radiologist in fracture assessment because the heat map typically does not contain information on the degree of fracture.
Localization and labeling of vertebra	[7–15]	In addition to the fracture identification and localization the algorithm also assigns the vertebral label such as T8 or L4 to each fractured or all vertebrae visible in the images. Labeling is typically an initial step of fully automated fracture detection and classification algorithms. Techniques like a support vector machine (SVM), random forest classification or CNNs are used for this task.
Segmentation	[7, 12–19]	AI-based automated vertebral fracture detection algorithms often require an automatic segmentation of the vertebrae or the vertebral bodies. Resulting segmentation masks facilitate the quantification of fracture. The masks can further be used to define the volume of interest for the measurement of BMD.
Vertebral morphometry and shape analysis	[20–22]	Morphological properties and shape models facilitate the determination of fracture grade and potentially the differentiation of osteoporotic from traumatic fractures and from degenerative deformities. Compared to 6-point X-ray-based morphometry a 3D shape analysis provides additional information, but it is not clear whether this information can be used to improve fracture risk prediction.
Grading of fracture severity	[19, 20, 23]	Severe vertebral fractures are associated with a higher risk of subsequent fractures than mild vertebral fractures. Thus, the determination of fracture grade is an important aim of vertebral fracture assessment.
Combinations	[15, 19, 24, 25]	Fully automated pipelines are increasingly being developed. They efficiently combine different algorithms for performing the tasks like vertebrae localization, labeling, segmentation, and classification to enable automatic fracture detection.
Classical techniques		
Segmentation	[26–31]	Classical algorithms developed to automatically segment the vertebral body or the complete vertebra. The resulting masks can be used for the same purposes as those generated with AI-based algorithms
Vertebral morphometry and shape analysis	[32, 33]	Classical algorithms to automatically determine vertebral morphometry and shape.
Grading of fracture severity	[34–36]	Morphological measures to classify vertebra fractures.

**Fig. 2:**
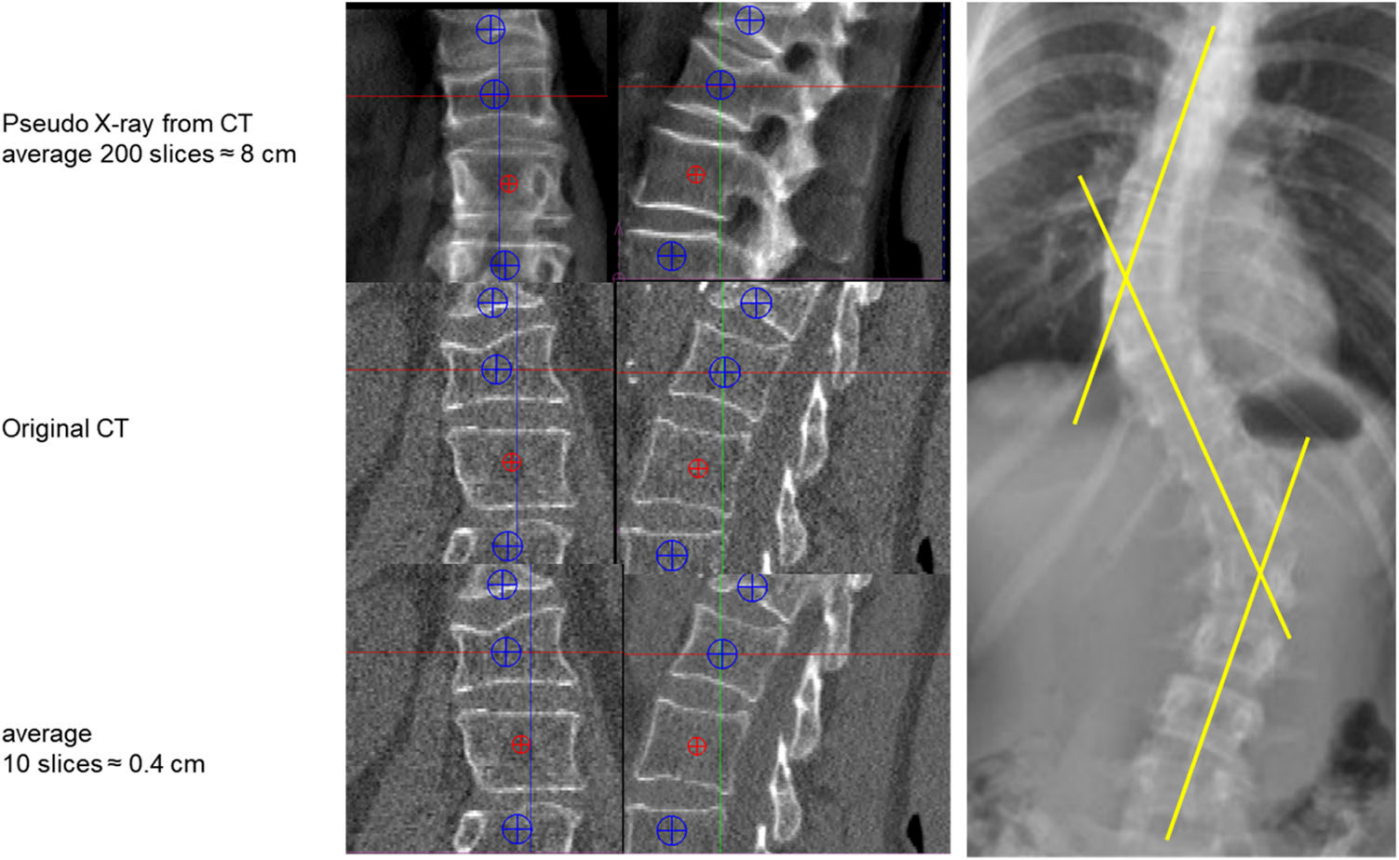
Left: coronal and sagittal reformations of CT dataset of the spine; top: simulated X-rays; center: original CT reformations; bottom: 10 slices averaged. Right: subject with sclerosis demonstrating that different coronal planes must be reconstructed from a CT dataset in order to assess mid-sagittal planes

Assessment of Genant scores from CT scans is challenging even for an experienced radiologist because CT scans provide 3D geometry and spatial resolution is lower than for spinal X-rays. Topics to consider are: (1) should coronal or sagittal CT reformations (or both) be used for spinal fracture assessment? (2) The use of single slices may easily result in overlooking fractures (Fig. 2) but how many slices should be averaged? In the case of scoliosis (coronal reformations) or kyphosis (sagittal reformations), the central slice of the set to be averaged may depend on vertebral level (Fig. 2). Also in contrast to X-rays, there is no atlas or extended description for CT to differentiate osteoporotic from traumatic fractures and from vertebral deformities unrelated to osteoporosis [41].

## Assessment of BMD

**Fig. 3:**
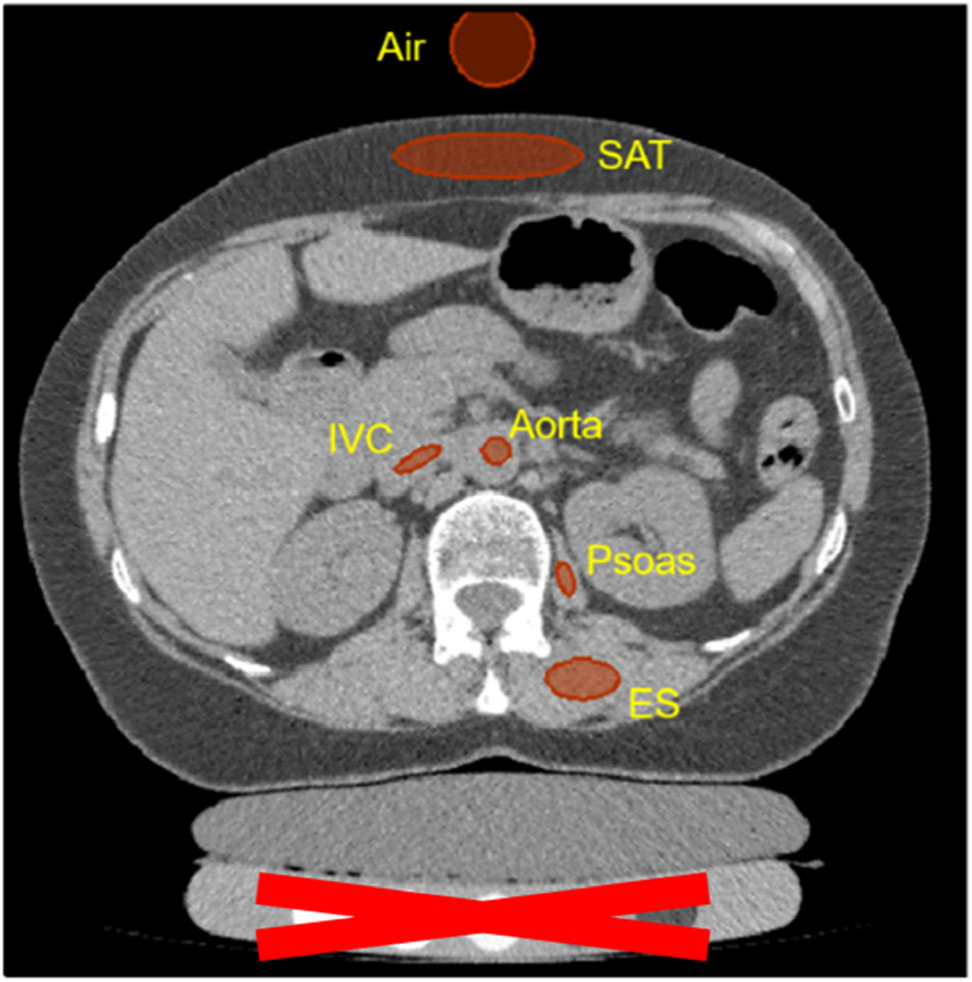
Volumes of interest used to determine CT values of tissues used for internal calibration: muscle of the erector spinae (ES) and the psoas muscle, blood in the aorta and the inferior vena cava (IVC), and subcutaneous adipose tissue (SAT)

In QCT the BMD is calculated from the measured CT values using a calibration phantom with inserts of known concentrations of hydroxyapatite or K_2_HPO_4_-water mixtures, which during the CT scan is positioned below the subject (simultaneous calibration) [1]. This procedure minimizes differences in BMD values across different CT scanner models. In the case of clinical CT, where such a calibration phantom is not used, three main options are available to assess BMD (Table 2). The first is the use of asynchronous calibration, a technique that separates subject and phantom scans [42, 65]. Depending on the stability of the CT scanner, the calibration phantom can be scanned once weekly or once monthly, for example. The second is called internal calibration as the phantom inserts are replaced by air and body tissues such as subcutaneous adipose tissue or blood (Fig. 3). As shown in Table 2, several internal calibration techniques have been proposed.

**Table 2:** Assessment of BMD without a calibration phantom measured simultaneously with the subject

Technique	Refs	Description	Pro and cons
Asynchronous calibration	[42–48]	Calibration phantom is not measured simultaneously with the subject but separately for example once a week or month. The CT value to BMD calibration procedure is the same as for simultaneous calibration.	+ X-ray field inhomogeneity effects can be avoided because the phantom can be scanned in the same location as the spine, for example, whereas in simultaneous calibration the phantom is positioned below the body - Scanner instabilities affecting the BMD calibration cannot be corrected if they occur between the patient and phantom scan - Workflow not implemented in a clinical routine yet
Internal calibration	[31, 49–57]	Use of air and body tissues such as muscle, blood, or subcutaneous adipose tissue for BMD calibration. Different approaches to calculate the linear calibration equation from CT values to BMD exist: 1. Calculation of ‘equivalent’ BMD values for the selected internal materials from a reference dataset using simultaneous calibration [49–54]. 2. Multiple linear regression between BMD values obtained from internal and from simultaneous calibration [31, 55, 56]. 3. BMD calibration based on the linear correlation between measured CT values of internal materials and their known density and absorption coefficients [57].	+ Scanner instability-related X-ray field inhomogeneity effects are smaller for internal calibration materials than for a calibration phantom positioned below the subject, as it is farther away from the bone for which BMD should be measured + Less affected by patient size than simultaneous calibration - Internal calibration is scanner and tube voltage specific - Methods 1 and 2 require a reference dataset with scans obtained on top of calibration phantom to derive the calibration equation and are not adequate for historic scans if such a dataset does not exist - Workflow not implemented in clinical routine yet
Direct use of HU values	[58–64]	No calibration equation used. Instead of BMD, CT values in HU units are used directly.	+ Most simplistic approach - CT scanner calibration to water does not guarantee identical HU values for hydroxyapatite - Thus, any HU threshold used instead of a BMD threshold will depend on the X-ray spectrum, i.e., on scanner, tube voltage, and potentially table height - All scanner instabilities directly affect CT values, which is not the case for simultaneous or internal calibration

The third option is the direct use of CT values in Hounsfield units (HU). A BMD calibration is not performed. The direct use of HU values requires scanner stability. HU values of bone depend on the energy distribution of the X-ray spectrum and are not normalized by the regular water calibration of CT scanners [66]; thus, HU values are scanner dependent as recently confirmed in a study of 67392 CT scans obtained from four different CT scanners [67]. Thus, further research on the use of CT values instead of BMD is warranted [68].

**Fig. 4:**
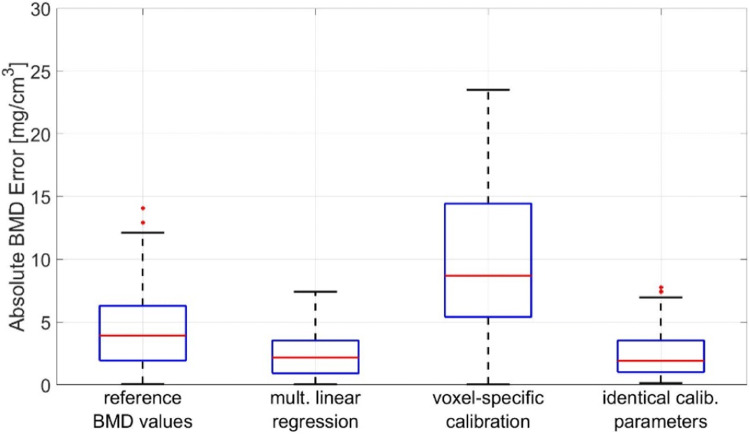
BMD calibration techniques applied in opportunistic screening compared to simultaneous calibration. 59 scans used for the analysis shown were obtained on a Siemens SOMATOM Definition AS scanner at 100 kV. Table heights varied by more than 10 cm across scans. Contrast agents were not administered

The accuracy of internal calibration is shown in Fig. 4. The comparison of techniques was performed using routine clinical CT scans that for research purposes also included a calibration phantom. For comparison, the fourth option shows the accuracy of using identical calibration parameters for all scans, equivalent to applying asynchronous calibration from a single phantom measurement. This procedure requires scanner stability, which was the case in this particular dataset but cannot be universally presumed for other datasets. In another dataset from clinical trials using highly standardized CT protocols, errors were about 50% lower compared to those shown in Fig. 4 [69].

It is interesting that several studies of opportunistic screening only used results from a single vertebra, most often L1 [58, 70–72], although it is recommended to use average results from at least two unfractured vertebrae, typically L1 and L2, to improve precision [1]. Phantomless BMD calibration methods for the spine can also be applied to abdominal or pelvic CT scans to determine hip BMD [43, 73].

Iodine-containing contrast agents are frequently administered prior to a CT scan in order to increase the contrast of blood-containing vessels and tissues. These contrast agents increase CT values [34, 43, 44, 74, 75]. The impact on BMD depends on the amount of contrast agent, i.e., the concentration within the bone of interest at the time of the CT scan. Cortical and trabecular bones compartments are affected differently. Also, the effect on spinal and hip BMD varies. In some studies using datasets with and without contrast of the same subject, linear regression correction techniques were developed [34, 45, 76, 77]; however, it is not entirely clear whether these techniques apply to the different scenarios in clinical routine and whether the linear correction factors obtained for one scanner can directly be used for different scanners and scan protocols [46]. Nevertheless, high correlations and good agreement between CT scans with and without contrast using linear corrections have been reported [74, 76, 77]. In the case of internal calibration, the change in the HU values of the internal reference materials after contrast administration remains to be studied [76]. Obviously, air that is often part of the internal calibration is unaffected by contrast agents.

## Performance of Opportunistic Screening

As mentioned in the introduction, the main purpose of OS is the identification of individual subjects with a high risk of osteoporotic fracture. It is less likely that OS will be applied in clinical trials unless historic CT scans should be used. In new studies, CT imaging will preferably be more standardized than routine clinical CT scans. If BMD is an endpoint, the use of a calibration phantom is advised. Thus the most important outcome of OS is fracture risk, which is also the most difficult to be used as a performance measure as it requires cross-sectional studies of fractured and unfractured subjects or ideally a prospective study such as AGES [78] or MrOs [79].

**Fig. 5:**
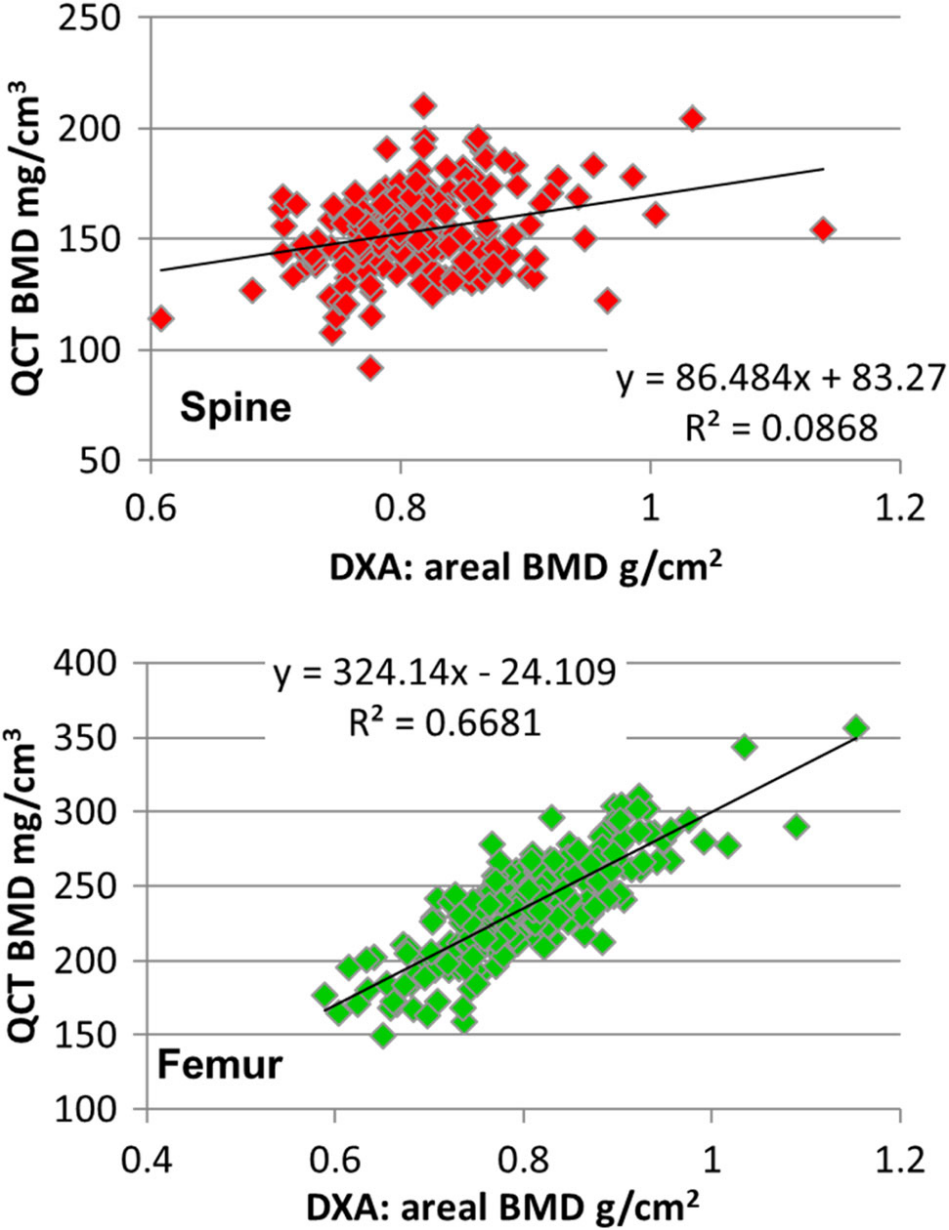
Correlation of DXA and QCT BMD of the lumbar spine (L1+L2) (top) and of the total femur. The graphs show baseline data from a clinical trial of postmenopausal women [86]. Correlations are moderate because the two techniques are different by nature

Instead, many studies evaluated the performance of OS by comparing the ability of CT and dual X-ray absorptiometry (DXA) to categorize subjects as normal, osteopenic, or osteoporotic using T-scores [59, 72, 80–84]. Conclusions are difficult to interpret as many subjects with DXA T-scores in the osteopenic or even normal range do fracture. Furthermore, the WHO schema is valid for DXA only but not for CT. DXA and CT BMD T-scores are not equivalent. Due to different risk gradients of the two modalities, a BMD-independent linear relation between DXA and CT BMD T-scores does not exist. The comparison of DXA and CT BMD values [85] is also misleading because due to the technical differences it is limited in particular for vertebral BMD (Fig. 5). The projectional DXA technique measures an areal density (aBMD) in g/cm^2^ and CT a true density in g/cm^3^ termed BMD in this contribution

Direct performance evaluation of BMD determined from CT scans not including a calibration phantom by asynchronous or internal calibration or of the direct use of HU values should be performed in a dataset of CT scans obtained with simultaneous calibration. It is also important to use a real-world CT dataset from clinical routine with variations of acquisition parameters, in particular kV and table height, instead of a dataset acquired with a highly standardized protocol. The same is true for evaluating the impact of contrast agents that are used in 50–70% of clinical CT scans. Performance evaluation should include scans with and without contrast of the same subject.

In a recent comprehensive multi-scanner study in which CT scans with and without phantom of the same subject were obtained within three days, BMD was used to discriminate vertebral fractures [87]. Performance of fracture discrimination was best for asynchronous, followed by simultaneous and non-calibrated BMD values, with AUC values of 0.86, 0.82, and 0.82, respectively. BMD as determined by QCT using simultaneous calibration correlated highly (*r*^2^ = 0.83) with BMD results from asynchronous BMD calibrations (slope 0.95). Nevertheless, 95% limits of agreement ranged from −23.2 to 25.0 mg/cm^3^. Given an approximate difference of 40 mg/cm^3^ between healthy and osteoporotic subjects [1], in individual subjects, the reported BMD differences may result in considerable over- or underestimation of their fracture risk.

In a 10-year longitudinal study of 199 subjects, CT values of L1–L4 were directly used to predict incident vertebral fractures (*n* = 30) as assessed from vertebral heights obtained from a mid-sagittal reformation of the CT dataset. Using a CT threshold of 180 HU for L4, incident vertebral fractures were predicted with a sensitivity of 90% and a specificity of 43%. A difference threshold of 155 HU for L5 resulted in a more balanced sensitivity (70%) and specificity (77%) [34] but also highlights ambiguities when using fixed thresholds for risk prediction.

Performance of vertebral fracture assessment is even more involved as ideally X-rays and CT scans of the same subject should be available, which is rarely the case. As an alternative, a validated radiological expert assessment of the CT scans could be used as the gold standard, where validation would require data on inter- and intra-reader comparison of expert results [88] including differentiation of osteoporotic from traumatic fracture and from other vertebral degenerations.

In 500 CT scans (50% with moderate or severe vertebral fractures) sensitivity and specificity of automated detection compared to expert radiological assessments were 94% and 65% respectively, but fracture severity grades were not determined. AUC results of HU values of L1 for the prediction of vertebral fractures were about 0.6 [89]. In another study of 150 subjects (50% with vertebral compression fractures) results of automated fracture detection compared to expert radiological assessments were 98.7% and 77.3% for sensitivity and specificity, respectively [20]. Similar values of 95% and 82% for sensitivity and specificity, respectively, were reported for the detection of any osteoporotic fracture [24]. This is one of the rare studies separating vertebral deformities from osteoporotic fractures. An AUC value of 0.74 was reported for vertebrae with mild fractures (Genant grade 1). It has also been shown that AI assistance improved the sensitivity of fracture detection by radiologists and non-radiologists [90].

Using CT scans of 48,227 CT scans from a health insurance registry it was shown that the combination of prevalent vertebral compression fractures with trabecular BMD of L1-L4 slightly improved the prediction of risk of major osteoporotic fractures (MOF) (AUC +1.9%, sensitivity +2.4%) and was equal for the prediction of hip fractures compared with the Fracture Risk Assessment Tool (FRAX). Both vertebral compression fractures and BMD were evaluated automatically using the Zebra Medical Vision toolkit [91].

Of course, a definite performance evaluation should include the determination of differences between OS and QCT/X-ray assessments on fracture risk. Given the aim to identify subjects with high fracture risk, a first goal could be the risk categorization as high, medium, or low, where medium would trigger additional diagnoses to more accurately determine the fracture risk. A high risk would indicate the need for intervention to be selected by an expert physician. A low risk would result in no further actions. For a more accurate risk assessment, relative fracture risk can be calculated from the assessment of prevalent vertebral fractures and form BMD results alone or in combination.

## Future Directions

A number of technological developments will further improve the performance of OS. With respect to fracture diagnosis, the refinement of AI models to obtain fracture grade and to clearly identify osteoporotic fractures is of key importance. Testing and validation of such models should be performed in different datasets, instead of just splitting a given dataset. At the same time, the criteria to assess these fractures from CT images need to be refined. Finally, new morphological or density features may be identified that further improve risk prediction of osteoporotic fractures.

With respect to BMD, further improvements partly rely on CT scanner technology. Obviously, the most simplistic approach would be the permanent addition of a calibration phantom, for example, embedded in the table of the CT scanner. However, the CT manufacturers have not yet recognized the potential of OS, so their focus on quantitative BMD determination is still limited.

**Fig. 6:**
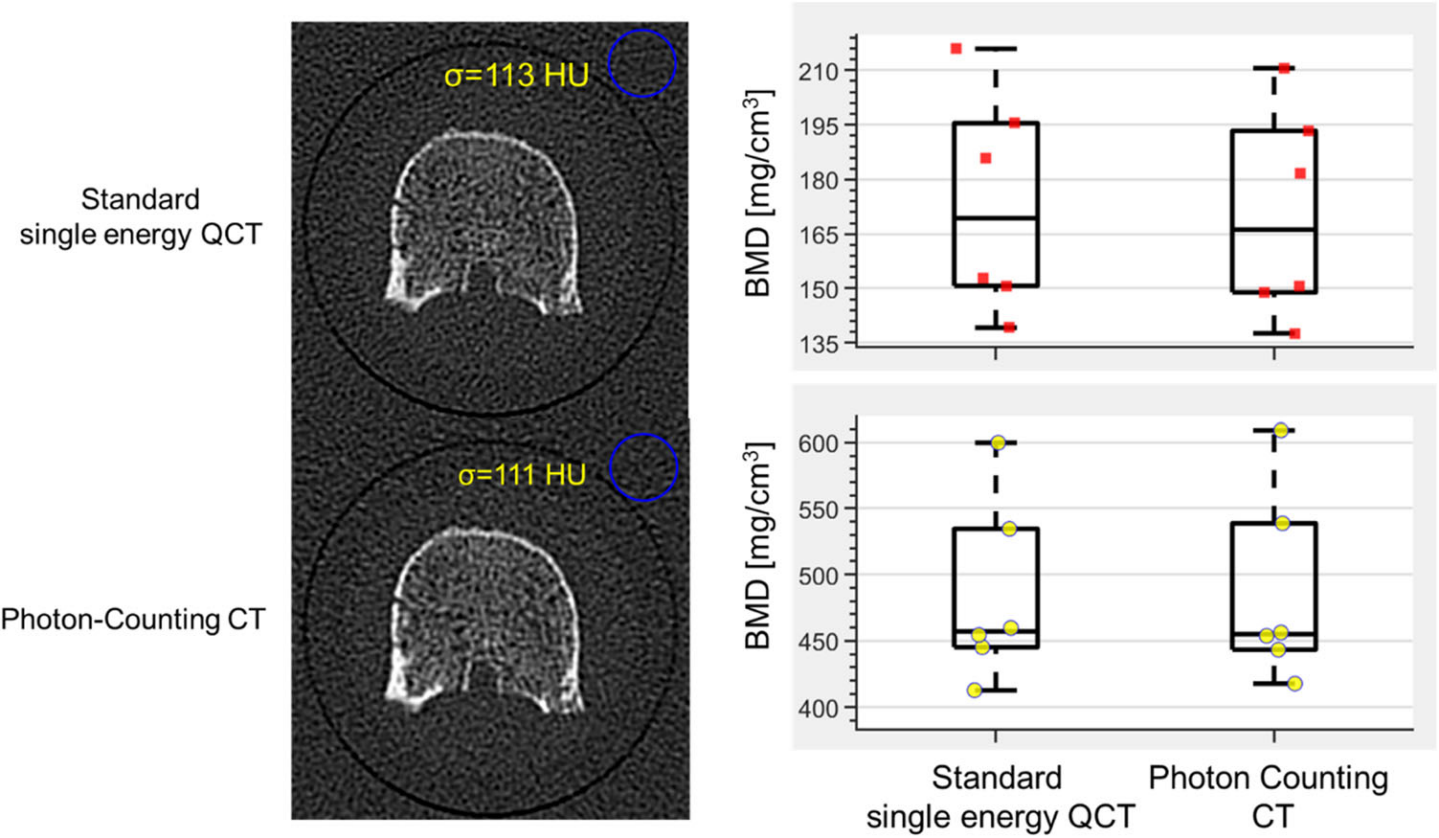
Dose reduction of photon counting CT using an excised vertebral body. Single energy CT: 120 kV, 355 mAs, 23.8 mGy, Photon counting 120 kV, 130 mAs, 10.5 mGy. The image noise (σ) is identical in both scans with a reduction of radiation exposure by more than 2 in the case of photon counting. For both scans, comparable high-resolution kernels were used [97]

Another perspective is the use of dual-energy CT that originally has been introduced for the reduction of the so-called fat error of single-energy CT [92–94]. Whether DECT could also benefit the accuracy of internal calibration still has to be investigated, first studies showed promising results [71, 77, 95, 96]. Also, the reduction of BMD inaccuracies caused by the administration of contrast agents should be a topic of future research. A further step will be the use of photon-counting CT scanners that will boost the use of CT in many areas because of several new features. One of the most important ones is the further reduction of radiation exposure. Figure 6 shows some very early results obtained on a prototype photon-counting device using excised vertebral bodies [97]. In this study radiation exposure was reduced by a factor of two without any degradation of image quality. Another important feature of photon counting CT is the further decomposition of the spectral response beyond the capabilities of DECT.

Finally, risk parameters other than prevalent fractures and BMD can be obtained from opportunistic CT scans to further improve fracture risk prediction. Examples are Finite Element Analysis to estimate bone strength [98, 99] and assessments of muscle size and density and fat infiltration that have been shown to contribute to fracture risk prediction beyond BMD [100–102]. Whether the assessment of spinal muscles or of the muscle of the hip is more important is not clear yet.

In summary, opportunistic screening can be successfully performed today. Now it is time for the implementation of the existing techniques into the clinical workflow of CT scanners to routinely identify subjects at high risk for osteoporotic fracture. Not all facets of opportunistic screening are fully automated yet and supervision of results is still required but fracture risk prediction can be further improved using advanced CT imaging and image processing techniques.
